# Community-engaged dissemination and implementation of an evidence-based health promotion intervention for Native American families: “Delivery of Turtle Island Tales to promote family wellness” protocol

**DOI:** 10.1186/s43058-025-00803-z

**Published:** 2025-11-20

**Authors:** Emily J. Tomayko, Alexandra K. Adams, Teresa Warne, James L. Merle, Paul A. Estabrooks

**Affiliations:** 1 Center for American Indian and Rural Health Excellence, Montana State University, Bozeman, MT 59718 US; 2 Department of Food Systems, Nutrition and Kinesiology, Montana State University, Bozeman, MT US; 3 Department of Population Health Sciences, University of Utah, Salt Lake City, UT US; 4 Department of Health and Kinesiology, University of Utah, Salt Lake City, UT US

**Keywords:** Native American populations, Poverty, Childhood obesity prevention, Dissemination and implementation science, Participatory action research, Evidenced-based interventions, SNAP-Ed programming, Program sustainability, Descriptive case study

## Abstract

**Background:**

Native American communities possess a wide range of assets that can contribute to reducing persistent inequities in food insecurity, obesity, cancer, chronic disease, and other related outcomes. Community engaged dissemination and implementation (CEDI) strategies that emphasize available, relevant, and generalizable evidence as well as community strengths and assets are well aligned to improve health outcomes with these communities.

**Methods:**

“Delivery of *Turtle Island Tales* to Promote Family Wellness” applies a culturally grounded, evidence-based intervention for obesity prevention through partnership with local organizations (e.g., Cooperative Extension/Supplemental Nutrition Assistance Program Education [SNAP-Ed]) to understand and enhance community capacity for sustained health promotion. A descriptive case study design applies bundled CEDI strategies (e.g., participatory Project Steering Committee; site-specific Community Implementation Teams) guided by the Consolidated Framework for Implementation Research and the Reach, Effectiveness, Adoption, Implementation, and Maintenance Framework to examine implementation across multiple communities. CEDI strategies will be tracked longitudinally, by community, to document iterative identification of locally specific and project general CEDI strategies as they relate to program reach, adoption, adaptation, implementation, and maintenance using mixed methods approaches (e.g., validated surveys, focus groups, interviews). An economic assessment of *Turtle Island Tales* also will be conducted.

**Discussion:**

This study applies innovative CEDI science to the equitable implementation of *Turtle Island Tales*, one of the only family-centered, home-based, evidence-based obesity prevention intervention developed for and with Native American communities. Key innovations include a mailed intervention model and culturally specific strategies that honor local community assets to support the program’s relevance, scalability, and long-term sustainability.

Contributions to the literature

- Describes a protocol to assess program implementation strategies to promote health and prevent chronic disease for Native American communities.
- Offers novel methods to maximize reach, adoption, and sustainability of culturally-tailored interventions, particularly in rural and frontier areas.
- Findings will inform the development of a Native American-specific community engaged dissemination and implementation strategy toolkit and an implementation research logic model to support the long-term delivery of health promotion and disease prevention interventions.
- Addresses important gaps in the body of literature regarding community engaged dissemination and implementation in Native American communities.

## Background

Native American communities encompass rich cultural traditions, strong family networks, and a deep connection to land and community, all of which serve as assets for promoting health and wellbeing. Despite these strengths, Native American communities experience disproportionately high rates of poverty and adverse health outcomes, including obesity, cancer, and chronic diseases, compared to non-Hispanic whites [1–4]. These differences in health outcomes emerge early in life, as Native American children also experience disproportionately high obesity rates [5–7], increasing their risk for cancers and chronic diseases in adulthood. As a result, Native American people have the lowest life expectancy of any population group in the US, with cancer as the leading cause of death for females and second leading cause for males [8].

Efforts to reduce these disparities have often relied on traditional health promotion models, such as classroom-based nutrition education or in-person cooking classes. However, these direct education formats may not effectively reach Native American families in rural, frontier, and persistent poverty areas, where geographic isolation, limited resources, and competing demands constrain participation [9]. Yet, few interventions are designed for the home environment, despite strong evidence supporting the critical role of the family setting in shaping children's health behaviors [10–13]. Even fewer family health promotion interventions have been co-developed with Native American families. The Supplemental Nutrition Assistance Program–Education (SNAP-Ed) Library of programs, for example, has limited home-based interventions or programs tailored to Native American communities. There remains a significant gap in culturally grounded, scalable, and sustainable interventions that address early-life health disparities for Native communities.

In response, many Native communities are revitalizing traditional practices that emphasize intergenerational knowledge sharing, sovereign food systems, and community-driven health initiatives. Strength-based approaches like these align closely with community-engaged dissemination and implementation (CEDI) strategies, which promote the use of available, relevant, and generalizable evidence while building on the strengths and assets of communities to enhance intervention adoption and reach [14]. The Healthy Children, Strong Families trials exemplified the use of CEDI strategies to design an early childhood obesity prevention intervention that prioritized characteristics likely to increase adoption and sustainment in Native American communities [15–18]. Co-developed with multiple Native American communities, the resulting home-based, family-centered intervention emphasized intergenerational wellness by promoting healthy eating, physical activity, sleep hygiene, emotional regulation, and other lifestyle factors. Based on positive findings from these trials, the program, later renamed *Turtle Island Tales*, was accepted into the SNAP-Ed Library as an evidence-based intervention. This action allows for *Turtle Island Tales* to be delivered using SNAP-Ed funds, but gaps remain in ensuring access and sustained implementation of the program, particularly in persistent poverty areas where typical, in-person health promotion strategies may be less effective.

By leveraging an innovative mailed, home-based program delivery model, *Turtle Island Tales* offers an alternative to the direct education format traditionally employed for SNAP-Ed programming. The project protocol described herein seeks to understand how to maximize reach, adoption, and sustainability of *Turtle Island Tales*, particularly in rural and frontier areas where access to in-person programming may be limited. To guide evaluation and implementation, the project applies the Consolidated Framework for Implementation Research (CFIR) [19] and the Reach, Effectiveness, Adoption, Implementation, and Maintenance (RE-AIM) framework [20] to increase the likelihood that culturally tailored interventions are systematically implemented and adapted within local community contexts. Coupled with CEDI strategies, this framework-guided approach aims to bridge the gap between evidence and practice in persistent poverty areas.

CEDI strategies in this study are intentionally aligned with Native American cultural strengths, such as engaging local grandmothers as program champions and incorporating community-specific language, foods, and traditions into intervention materials. Partnerships with SNAP-Ed implementing agencies (often Cooperative Extension programs at land-grant universities) and other local organizations across multiple states will allow for systematic evaluation of the program’s scalability and long-term sustainability in varied contexts. By fostering community ownership and integrating implementation science frameworks with local cultural practices, the project aims to advance sustainable, community-driven solutions to promote child and family wellness and reduce chronic disease risk. These efforts directly inform the overall study’s objectives to enhance reach, improve effectiveness, and evaluate implementation outcomes in the context of persistent poverty for rural Native American communities.

## Methods

### Project objectives

To achieve the central goal of supporting sustained implementation of *Turtle Island Tales* through community-informed CEDI strategies, the aims of this project include (1) supporting and evaluating the reach of *Turtle Island Tales* when delivered through Extension/SNAP-Ed in Native American communities experiencing persistent poverty, (2) evaluating the capacity of state-level Extension and SNAP-Ed systems to adopt, implement, and sustain the program over time, (3) determining the short- and long-term budget impact of integrating *Turtle Island Tales* into routine program delivery, and (4) capturing the different implementation strategies used across implementing sites and the dynamic changes made to strategies within and across each site over the course of program delivery.

### Study design and setting

This multilevel participatory action research project will use a descriptive case study methodology across four state SNAP-Ed programs that will be engaged to facilitate the dissemination, implementation, and sustainability potential of *Turtle Island Tales*. In many states, Cooperative Extension programs of land-grant universities serve as SNAP-Ed implementing agencies; therefore, we will engage partners working within both systems. CFIR and RE-AIM will be integrated to systematically address implementation barriers and promote the co-creation of sustainable dissemination strategies, with reach as the primary outcome. A convergent mixed methods approach will combine quantitative assessments related to predictors from the CFIR and RE-AIM outcomes with qualitative data to better characterize the planning, execution, and evaluation of CEDI strategies.

**Fig. 1:**
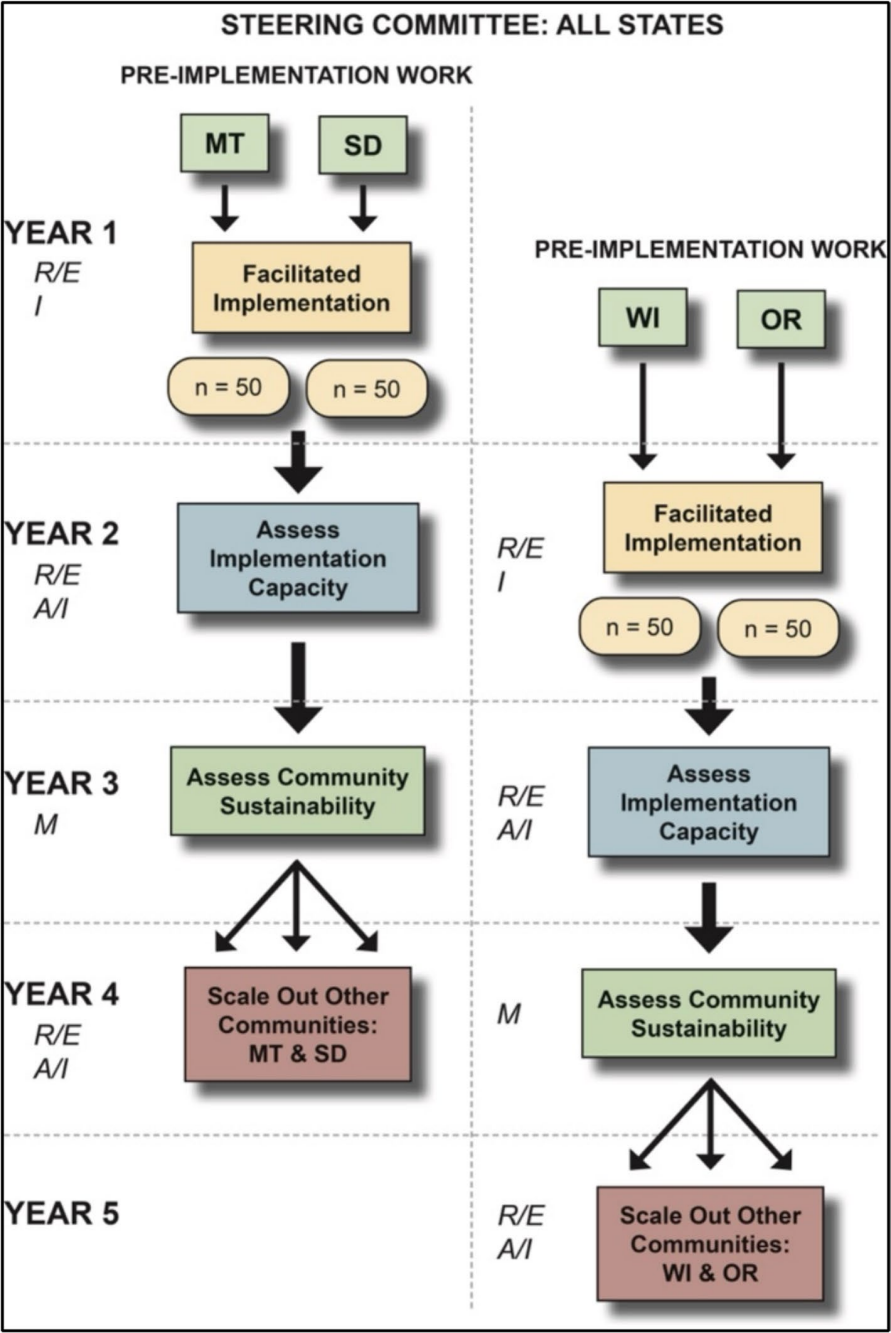
Staggered implementation timeline. MT, Montana; SD, South Dakota; WI, Wisconsin; OR, Oregon. RE-AIM, reach, effectiveness, adoption, implementation, maintenance

This project will be conducted in SNAP-Ed systems across four states: Montana, Oregon, South Dakota, and Wisconsin. All participating census tracts are rural, with 43% classified as frontier. Local SNAP-Ed agents and Native American community partners will lead the implementation efforts, focusing on state-specific community partnerships with reservation-based Native American communities. We anticipate that each Community Implementation Team will include local SNAP-Ed agents, tribal partners, and other community representatives. Figure 1 outlines the five-year, staggered implementation and evaluation timeline for *Turtle Island Tales* across four planned states. Under the guidance of the shared Project Steering Committee, Montana and South Dakota will begin with pre-implementation work in Year 1, followed by facilitated implementation with 50 families per state. Implementation capacity in these state systems will be assessed in Year 2, followed by community sustainability evaluation in Year 3. After the initial implementation year, we will track scale-out to additional communities within each state. Wisconsin and Oregon start pre-implementation work in Year 1, begin facilitated implementation in Year 2, and proceed through the same phased evaluation process in Years 3 to 5. Key evaluation outcomes, aligned with the RE-AIM framework, are embedded throughout all phases.

### Participants

Recruitment will occur through SNAP-Ed personnel in collaboration with local Community Implementation Teams formed within each participating community. The Community Implementation Teams will guide recruitment strategy selection based on prior local effectiveness and community preferences. Community Implementation Teams will select families with children ages 3–8 years old residing in persistent poverty areas to participate in the year-long *Turtle Island Tales *program as part of standard SNAP-Ed nutrition education delivery. Our prior work indicated Facebook posts and direct engagement through Head Start programs were particularly effective in reaching families [15, 21]. Each site will aim to enroll up to 50 families during the program’s first year, with strategies tailored to the community’s needs and resources. Program delivery staff also will participate in program evaluation components conducted through Community Implementation Teams and the broader, cross-state Project Steering Committee.

### Intervention description

*Turtle Island Tales *is an evidence-based obesity prevention intervention that aims to reinforce Native American cultural values of family interaction and holistic wellness [15–18]. This home-based program targets six health behaviors: increase fruit/vegetable intake, decrease added sugar intake, increase physical activity, decrease sedentary/screen time, promote healthy sleep, and promote emotional regulation. The year-long, 12-lesson program is designed to be mailed into the home monthly as a kit that contains the following core intervention components: printed educational lessons for adults, a commercially available children’s book on the topic, coloring sheets, goal setting cards, recipe cards, support items, and family activity ideas. A Native Advisory Group provided traditional knowledge for the program, in keeping with Native values of elders passing on knowledge for the next seven generations. Core process components of the intervention include monthly mailing of kits into the home, a grandmother from within the community to champion the program, a cohort structure to start in alignment with school/Head Start schedules, and social media support.

### CEDI strategies

Several bundled strategies are proposed to improve reach (primary outcome), adoption, implementation, and maintenance of program delivery (Table 1). The proposed strategies will support the local identification, adaptation, or creation of additional local strategies. These bundled strategy approaches were informed by a number of sources, including those used during the original development of *Turtle Island Tales*, the Expert Recommendations for Implementing Change compilation [22], community-based participatory research principles, and strategies mapped to CFIR constructs hypothesized to influence RE-AIM outcomes. Anticipated bundled strategies include: (1) multi-leveled engagement within and across communities through a Project Steering Committee and site-specific Community Implementation Teams, (2) use of an evidence-based, community co-produced intervention (*Turtle Island Tales*) with an implementation blueprint, and (3) centralized distribution with technical assistance for Community Implementation Teams. While these strategies were proposed a priori, additional strategies will be collaboratively identified and refined by the Project Steering Committee and Community Implementation Teams throughout the study. Specifically, we expect that strategies such as fund and contract, facilitation to support initial implementation and sustainment, and development of tools for quality monitoring will be discussed and selected as implementation progresses (Table 1).

**Table 1:** Specified strategies intended to increase the reach, adoption, implementation, and maintenance of *Turtle Island Tales*

CEDI strategy	Actor(s)	Action(s)	Intended audience of Action(s)	Temporality	Dose	Intended CFIR mechanism(s)	Intended RE-AIM outcome(s)
Multi-leveled engagement with advisory boards, members from the population intended to benefit, and previous family participants	Academic & community partners, champions, decision makers & doers	Collaborative planning, development, and evaluation of a culturally relevant, family-based obesity prevention intervention	Native American families	Research question and intervention development, testing, and interpretation of results	Monthly advisory board meetings, 36 months; five focus groups with program participants; two randomized controlled trials	*Intervention Characteristics*; source, cultural compatibility, relative advantage, complexity	Reach, Effectiveness, Adoption, Implementation, Maintenance (individual and program)
Co-production, intervention packaging, and developing a formal implementation blueprint	Native American Communities, Research Team, CITs	Collaborative development of culturally relevant, family-based obesity prevention intervention	Native American families	Intervention development, testing, and interpretation of results	Within monthly CIT meetings; use of blueprint across facilitation strategies	*Intervention Characteristics*; source, cultural compatibility, relative advantage, complexity; design quality and packaging	Reach, Effectiveness, Adoption, Implementation, Maintenance (individual and program)
Centralize distribution and technical assistance (dissemination organization)	Research Team	Distribute *Turtle Island Tales* to families recruited in local communities	SNAP-Ed providers; other community implementors	During implementation, Sustainment, Scale-out	Monthly; technical assistance as needed	*Intervention Characteristics*; design quality and packaging *Implementation Process*; Facilitation	Reach, Adoption, Implementation
Fund & contract	Research Team (project budget)	Provide initial funding support for *Turtle Island Tales* in each community	SNAP-Ed Program Implementors, Administrators	Pre-implementation	Once	*Inner setting;* Implementation climate, compatibility, available resources *Outer setting; external policy and incentives*	Adoption, Implementation
Facilitation to support initial implementation and sustainment	Research Team, CITs	Community-specific problem solving, budget, development, and submission support specific to SNAP-Ed and other available resources	SNAP-Ed Program Implementors, Administrators	Pre-implementation, Implementation, Sustainment, Scale out	Ongoing, monthly, 5 years	*Inner setting;* Implementation climate, compatibility, available resources *Implementation Process;* Planning, executing, reflecting & evaluating	Reach, Implementation, Maintenance (program)
Develop and implement tools for quality monitoring	Research Team, CITs, PSC	Use SNAP-Ed systems to create ongoing monitoring of implementation and outcome measures	SNAP-Ed Program Implementors, Administrators	Pre-implementation, Implementation, Sustainment, Scale out	Ongoing in PSC & CIT meetings (5 years)	*Inner setting;* Implementation climate *Outer setting; external policy and incentives*	Implementation, Maintenance (program)

*Acronyms:* *CAIRHE* Center for American Indian and Rural Health Excellence, *CEDI* Community Engaged Dissemination and Implementation, *CFIR* Consolidated Framework for Implementation Research, *CIT* Community Implementation Team, *PSC* Project Steering Committee, *RE-AIM* Reach, Effectiveness, Adoption, Implementation, and Maintenance Framework, *SNAP-Ed* Supplemental Nutrition Assistance Program Education

All implementation strategies, whether proposed in advance or developed during the study, will be iteratively identified using the Longitudinal Implementation Strategies Tracking System (LISTS) methodology and online data capture tool (https://hivimpsci.northwestern.edu/longitudinal-implementation-strategy-tracking-system/) [23]. Within the LISTS tool, strategies are documented according to specifications from Proctor et al. [24] and categorized using the Expert Recommendations for Implementing Change taxonomy [22]. Modifications to strategies are captured using methods from the Framework for Reporting Adaptations and Modifications to Evidence-Based Implementation Strategies (FRAME-IS). Adaptations will be tracked within LISTS using FRAME-IS criteria to record the actor, action, temporality, dose, planned/unplanned nature, and rationale. This ensures systematic documentation of changes in both strategies and implementation protocol. LISTS will allow us to track dynamic changes to both strategy specification and the implementation protocol (e.g., strategies added, strategies discontinued, whether changes were planned, and why changes were made) that occur during our project in a valid and reliable manner by including a routine timeline follow-back procedure.

In addition, the Project Steering Committee and Community Implementation Teams will co-develop an Implementation Research Logic Model to guide future CEDI work in similar contexts [25]. Strategies considered successful will be compiled into a CEDI Strategy Toolkit to support RE-AIM outcomes for *Turtle Island Tales* and related interventions for Native communities.

## Data collection

Quantitative data will include program reach metrics (e.g., proportion of enrolled families vs. eligible families) and dissemination and implementation strategy process tracking using LISTS (Table 2). Fidelity to program delivery will be assessed during monthly Community Implementation Team meetings in collaboration with the research team. These meetings will review records of monthly kit mailings, focusing on delivery timing and completeness to ensure adherence to the intervention protocol and support continuous quality improvement. Qualitative data will be gathered through transcripts from quarterly Project Steering Committee and Community Implementation Team meetings, focus groups, and interviews​. Effectiveness of the program to impact health behavior change and food security will be measured under a separate study protocol (NCT06298149).

**Table 2:** Quantitative outcome measure summary

Construct	Measure	Source(s)	Collected By
**RE-AIM measures**			
Reach	Number/proportion of Native American families in persistent poverty areas who receive *Turtle Island Tales;* representativeness based on race, ethnicity, economic status	Number of kits delivered	Research Team
Adoption	Number/proportion of Native American communities within given state that deliver *Turtle Island Tales; r*epresentativeness based on communities in persistent poverty areas	State-level reports	SNAP-Ed
Implementation: *Fidelity*	Proportion *Turtle Island Tales* toolkits delivered as planned	Monthly checklist with CITs	Research Team
Implementation: *Cost*	Material costs; implementation time; ongoing costs	Monthly checklist with CITs	Research Team
Maintenance	Funding stability; administrative support; partnerships; organizational capacity	CIT survey; Program Sustainability Assessment Tool	Research Team
**Consolidated Framework for Implementation Research measures**			
Inner setting	Implementation climate, compatibility, available resources	CIT Survey	Research Team
Characteristics of individuals involved	SNAP-Ed agent knowledge, self-efficacy	CIT Survey	Research Team
Outer setting	Cosmopolitanism (SNAP-Ed networked with relevant community organizations), external policy & incentives	CIT Survey	Research Team
Process of implementation	Key stakeholders, opinion leaders, planning, executing, reflecting & evaluating	CIT Survey	Research Team

Effectiveness (food security, fruit/vegetable intake) will be measured under a separate protocol

*Acronyms:* *CIT* Community Implementation Team, *RE-AIM* Reach, Effectiveness, Adoption, Implementation, and Maintenance Framework, *SNAP-Ed* Supplemental Nutrition Assistance Program Education

## Analysis

To assess program reach, we will track the number and proportion of families enrolled in *Turtle Island Tales* across participating communities. Representativeness will be evaluated using odds ratios (90% confidence interval) comparing enrolled families to relevant community census data (e.g., poverty status, age, sex). Recruitment rates will be analyzed using the number of families exposed to recruitment as the denominator, with time to enrollment of 25 and 50 families calculated per site. Differences in enrollment success across recruitment strategies will be assessed using the *prop.test *function in R, and community-level reach trends will be visualized relative to the timing of strategy implementation. Qualitative data from meetings and monthly feedback surveys will undergo descriptive content analysis [26], with transcripts double-coded in NVivo by two trained coders. In addition to descriptive content analysis, all Project Steering Committee and Community Implementation Team meetings will be transcribed with coding guided by CFIR and RE-AIM. This will allow the research team to summarize thematic content efficiently, identify emerging patterns across sites, and generate community-oriented recommendations.

To evaluate adoption, implementation, and maintenance, we will apply a convergent mixed methods approach, combining quantitative predictors from the CFIR and RE-AIM frameworks with qualitative data collected in an ongoing, parallel manner. Both data types will be equally valued, and mixed methods analysis will follow established implementation research procedures [27]. Quantitative analysis will include a descriptive summarization of CFIR domains over time and across sites, with pairwise comparisons to assess changes. Short-term budget impact analysis will capture costs associated with adoption and implementation during the study period. To project long-term sustainability, we will forecast five-year budgetary trends using the Bureau of Labor Statistics Employment Cost Index for staff costs and the Consumer Price Index for material and mailing costs. Community Implementation Team members will report on hours spent on implementation tasks (excluding research-specific activities). Personnel costs will be estimated using market-based salaries, and material costs will be based on invoices. Trendlines for anticipated cost growth will be developed to support planning for future program delivery.

The LISTS tool provides both visual and tabular data outputs that will be critically appraised to describe the implementation process across each implementing site, including the number and categorization of strategies used, modifications made, whether these modifications were planned, which elements were modified (actor, action, target, dose), and a justification [28]. LISTS data will also be used to deepen the quantitative analysis on the primary outcome of reach to conduct various sensitivity analyses to contextualize the main effects. We will also utilize the Implementation Research Logic Model to organize the relationships among implementation determinants, strategies, and their purported primary and secondary outcomes.

## Discussion

This study addresses the critical need to understand implementation of culturally relevant, evidence-based interventions to prevent obesity and reduce cancer risk in Native American communities, particularly those experiencing persistent poverty. The "Delivery of *Turtle Island Tales* to Promote Family Wellness" study leverages innovative CEDI strategies to promote broad reach and sustainable implementation within SNAP-Ed systems across four states. Although childhood obesity prevention is recognized as a strategy to reduce lifetime cancer and chronic disease risk, few interventions have been developed to reflect the unique cultural and contextual realities of Native American families or to reach them at scale. Successful implementation often depends on trusted community champions—for example, respected elders—and tailoring materials to specific language and cultural contexts.

This study employs rigorous, theory-driven methods, including the RE-AIM and CFIR frameworks, to systematically address barriers and evaluate implementation processes across multiple ecological levels. A novel feature is the introduction of a mailed intervention approach within SNAP-Ed programming, which represents a significant departure from the direct education methods typically employed by SNAP-Ed. Given changes in service delivery following COVID-19, SNAP-Ed agents have expressed strong interest in expanding this model to increase program reach and sustainability. Moreover, tracking the dynamic nature of implementation strategy use with the LISTS method is a novel contribution that will rigorously evaluate the complex CEDI process. These CEDI strategies primarily focus on the CFIR construct of intervention characteristics, including the intervention source, compatibility with SNAP-Ed delivery systems and participant lifestyles, low implementation complexity, and relative advantage. Through detailed tracking of implementation strategies and corresponding CFIR constructs, we plan to identify how specific mechanisms, such as improved cultural compatibility, increased perceived relative advantage, and reduced complexity, contribute to improvements in RE-AIM outcomes with a primary focus on reach. These hypothesized mechanisms are expected to guide understanding of how contextually aligned CEDI strategies lead to improved RE-AIM outcomes of *Turtle Island Tales*.

In consultation with our community and SNAP-Ed partners during the study planning phase, we considered alternative designs, including randomization of communities to different implementation strategies or quasi-experimental comparisons but determined these approaches were not feasible. Limited foundational knowledge exists about which CEDI strategies are most effective for scaling out obesity prevention interventions in Native American communities, and delaying program support could discourage future program adoption. SNAP-Ed programs and community partners with interest in *Turtle Island Tales* are unlikely to accept assignment to waitlist or comparison conditions, given competing demands and pressing community needs. Thus, a pragmatic, mixed-methods case study design was selected to both advance implementation science and support timely, meaningful local impact. Although analytic approaches such as Cox hazard models were considered to examine differences in reach, they were deemed inappropriate given the non-randomized, partner-driven design and anticipated sample sizes.

An important contextual consideration for this study is the budgetary limitations inherent in SNAP-Ed and other public health programs. Within SNAP-Ed, allowable costs are typically directed to direct education efforts, with minimal resources for indirect education, capacity building, or maintenance activities. Consequently, identifying cost-effective, adaptable implementation strategies is critical for ensuring that interventions like *Turtle Island Tales* can be maintained once external research support concludes, particularly given that so few culturally relevant interventions are available for young Native American children. This project addresses these realities by embedding cost-tracking and sustainability planning across all phases of implementation, with the goal of producing practical, scalable solutions for programs operating in rural/frontier and persistent poverty areas.

Moreover, the “Delivery of *Turtle Island Tales* to Promote Family Wellness” study will systematically document and characterize culturally and locally tailored CEDI strategies. Because they will be co-produced with community partners, these strategies are expected to influence health outcomes across varied settings and enhance understanding of how to support long-term program success in real-world environments. The project’s conceptual framework integrates social ecological, dissemination and implementation, and behavioral theories, offering a model for addressing health promotion that is responsive to the lived experience of Native families. Further, the use of convergent mixed methods will enable us to examine cross-site variation in implementation, propose adaptable versus core components of CEDI strategies, and inform generalizability of findings to other Native American and rural communities.

Despite co-production of CEDI strategies and ongoing support, it is possible that some communities may face unanticipated barriers to adoption, implementation, or sustainment. Factors such as staff turnover, shifting administrative priorities, or competing local initiatives may affect program continuity. Our study design accounts for this by documenting site-specific adaptations and progress, which are critical to refining our CEDI strategy toolkit for future applications. Our findings will directly inform the development of a Native American-specific CEDI strategy toolkit and an implementation research logic model to support the long-term Delivery of *Turtle Island Tales* and similar interventions aimed at health promotion and disease prevention. Ultimately, outcomes from this study aim to guide future efforts to adapt, scale, and sustain culturally grounded interventions across a broader range of communities and SNAP-Ed systems nationwide, contributing to efforts to strengthen public health infrastructure and intervention impact.

## Data Availability

Data will be available upon reasonable request.

## Abbreviations

CEDI: Community engaged dissemination and implementation
SNAP-Ed: Supplemental Nutrition Assistance Program Education
CFIR: Consolidated Framework for Implementation Research
RE-AIM: Reach, Effectiveness, Adoption, Implementation, and Maintenance
LISTS: Longitudinal Implementation Strategies Tracking System
FRAME-IS: Framework for Reporting Adaptations and Modifications to Evidence-Based Implementation Strategies
